# A double-edged impact of AI literacy on consumers’ willingness to use AI-powered products: a parallel mediation model of perceived AI risk and perceived social good

**DOI:** 10.3389/fpsyg.2026.1967967

**Published:** 2026-09-21

**Authors:** Yuxiang Na, Shiqiang Liu, Xin Yan

**Affiliations:** 1 School of Journalism and Communication, Jinan University, Guangzhou, China; 2 International School, Jinan University, Guangzhou, China

**Keywords:** AI literacy, AI use, perceived risk, social good, value-based adoption model

## Abstract

**Introduction:**

The widespread adoption of artificial intelligence technologies has driven sustained growth in consumer demand for AI-powered products. However, the role of AI literacy as a key determinant of consumers’ willingness to use such products remains subject to conflicting conclusions regarding its underlying mechanisms.

**Methods:**

This study compared medical and conversational scenarios, constructing a parallel mediation model based on the Value-based Adoption Model. This model featured perceived AI risk and perceived social good as two opposing mediators, exploring the influence mechanism of AI literacy on consumers’ willingness to use AI-powered products.

**Results:**

Our survey of 441 consumers showed that higher AI literacy significantly increases consumers’ willingness to use chat-based AI and medical AI. Perceived social good and perceived AI risk mediated the relationship between AI literacy and willingness to use chat-based AI. The positive mediating effect of perceived social good (*b* = 0.32, 95% CI [0.19, 0.45]) was stronger than the negative mediating effect of perceived AI risk (*b* = −0.03, 95% CI [−0.07, −0.003]). Perceived AI risk mediated the relationship between AI literacy and consumers’ willingness to use medical AI (*b* = −0.08, 95% CI [−0.16, −0.01]), while the mediating effect of perceived social good was not significant (*b* = 0.04, 95% CI [−0.11, 0.17]).

**Discussion:**

This study revealed a dual-path mediation mechanism through which AI literacy influences consumers’ willingness to use AI-powered products, as well as its cross-scenario heterogeneity, which not only explained the inconsistent findings in prior research but also provided practical insights for scenario-based deployment of AI-powered products and the cultivation of consumer AI literacy.

## 1 Introduction

While artificial intelligence (AI) is fundamentally reshaping social structures and the boundaries of consumer life, it simultaneously manifests a pronounced technological duality: it offers unprecedented convenience and efficiency while exposing consumers to risks such as AI hallucinations, algorithmic bias, and privacy erosion. Within this context, AI literacy—defined as individuals’ capacity to understand, apply, and critically evaluate AI systems, as well as to reflect on their ethical, social, and legal implications—has emerged as a core consumer competence in the intelligent marketplace (Almatrafi et al., 2024; Sperling et al., 2024).

However, the psychological and behavioral consequences of AI literacy are far from straightforward. Existing empirical findings reveal substantial heterogeneity. Some studies demonstrate that higher AI literacy enhances trust in AI systems and strengthens adoption intentions (Wang et al., 2025; Zhao, 2025). In contrast, some studies document an “acceptance paradox,” whereby consumers with lower AI literacy are more willing to adopt AI technologies (Li et al., 2026; Tully et al., 2025). This divergence challenges the assumption that improving literacy straightforwardly promotes technology acceptance and complicates policy efforts aimed at literacy-driven consumer empowerment.

Furthermore, previous research has primarily examined AI literacy from an educational perspective, focusing on teachers and students. Empirical studies on adult consumers’ AI literacy, particularly regarding its psychological mechanisms and behavioral consequences across different application scenarios, remain relatively scarce. Consequently, despite growing academic attention in this field, numerous unresolved debates and empirical gaps persist—especially concerning contextual variations and mechanistic explanations.

Based on the above, this paper will focus on the inconsistent effects of AI literacy observed in previous studies and transcend the limitations of single-scenario analyses. By comparing two representative yet heterogeneous AI application scenarios—healthcare and social chat—it systematically examines the influence of AI literacy on consumers’ behavioral intentions and its underlying mechanisms. To this end, this study employs the Value-based Adoption Model (VAM) as its theoretical framework. It constructs a parallel mediation model with AI literacy as the independent variable, willingness to use AI as the dependent variable, and introduces perceived AI risk and perceived social good as two parallel mediating variables that may have competing effects. By empirically testing the mediating effects and strength differences of these perceptions across diverse scenarios, this study aims to provide an integrated explanatory perspective on the complex mechanisms of AI literacy. This approach thereby offers theoretical reference points and practical insights to deepen the contextual adaptation and consumer-oriented marketing of intelligent technologies.

## 2 Theoretical background and hypotheses development

### 2.1 From media literacy to AI literacy

Throughout the course of technological advancement, academic research has consistently focused on how humanity leverages technology to empower social life. Early scholars introduced the concept of “media literacy,” aiming to systematically examine the interactive relationship between individuals and media technologies. Media literacy is understood as “the knowledge, skills, and competencies required to use and interpret media” (Buckingham, 2015). As early as the 1930s, the United Kingdom began advocating for media literacy education, initially aimed at countering the popular cultural values disseminated by mass media at the time. With the evolution of media formats and the increasing complexity of the technological environment, concepts such as “information literacy” and “digital literacy” emerged successively, further expanding the academic focus on the public’s ability to navigate information and engage in digital practices within emerging technological landscapes (Koltay, 2011).

As we enter the age of intelligence, the advancement of artificial intelligence technology has propelled the theoretical paradigm of media literacy to continue evolving, with academia gradually shifting its focus toward the construction and exploration of “AI literacy.” Existing research views AI literacy as an advanced form of media literacy within the context of intelligent technology (Ali et al., 2026), encompassing multiple dimensions such as human-machine collaboration, interpersonal communication, ethical judgment, and risk management. As AI products and services become deeply embedded in public life, AI literacy concerns not only individuals’ capacity for critical evaluation of artificial intelligence technology but also effective interaction between humans and technological systems, along with their practical application efficacy in societal contexts. Previous research examined how AI literacy influences public perception and usage of AI products and services. For instance, Al-Abdullatif and Alsubaie (2024) noted that users with a clear understanding of algorithmic principles and functional boundaries can more efficiently integrate AI tools into learning and work contexts. Labrague et al. (2023) found in their study of nursing students that students’ self-assessed technical competence, level of understanding of artificial intelligence, and recognition of AI’s potential applications in nursing significantly influenced their willingness to use AI. These empirical findings confirm the critical role AI literacy plays in mediating human-machine interactions and empowering individual actions.

### 2.2 AI literacy and willingness to use AI products

Artificial intelligence literacy, as a key indicator of individual adaptability in the intelligent era, has become a core topic of interdisciplinary interest across communication studies, information science, and management. Existing research has extensively explored its influence across diverse application scenarios and consumer groups. In higher education research, AI literacy exerted a significant positive impact on doctoral students’ innovative behaviors (Weikang et al., 2025). Research in the field of education found that middle school students’ AI literacy could positively predict their future interest in pursuing AI-related careers and their intention to learn AI knowledge. This conclusion provides empirical evidence for systematically introducing artificial intelligence education at the primary and secondary school levels (Zhang D. et al., 2025). In healthcare settings, a survey of Korean medical students indicated that AI literacy was significantly positively correlated with their willingness to utilize AI-assisted diagnosis and treatment in clinical practice (Si, 2025). The aforementioned studies collectively reveal that AI literacy positively influences consumers’ technological cognition, attitudes, and usage intentions across diverse social practices, further underscoring the critical importance of cultivating consumers’ AI literacy.

Notably, while existing literature generally confirms that AI literacy exerts positive effects on individual cognition and behavior, some empirical findings present contradictory results. For instance, Li et al. (2026) demonstrated through two situational experiments that AI literacy may inhibit technology acceptance. Their study revealed that consumers with lower AI literacy exhibited higher tendencies toward adopting AI technologies, with this relationship mediated by feelings of mystique and awe. Similarly, Tully et al. (2025) found that consumers with lower AI literacy tend to be more receptive to AI technology. This counterintuitive association does not stem from differences in the consumers’ AI capabilities or ethical awareness, but rather from the fact that individuals with lower AI literacy are more likely to experience awe toward AI technology, thereby increasing their acceptance.

Overall, despite these heterogeneous findings, most studies still support the positive impact of AI literacy. Higher literacy levels generally correlate with stronger consumer acceptance, trust, and willingness to use AI technologies and related services. Considering current human-machine interaction practices, medical AI and chat-based AI represent two key domains where consumers encounter and utilize intelligent technologies. In healthcare settings, artificial intelligence is integrated throughout the healthcare management process—from image recognition, diagnostic assistance, and treatment value or public health benefits evaluation to disease progression tracking. Its technological applications have demonstrated significant social efficacy and clinical translation potential (Doumat et al., 2022). Meanwhile, in social settings, artificial intelligence is gradually permeating numerous everyday domains such as cultural tourism communication, education and teaching, emotional companionship, and intelligent customer service, serving as key interactive tools (Boloș et al., 2024; Park et al., 2025). Therefore, this study focuses on medical and conversational AI applications to explore the mechanism by which consumers’ AI literacy influences consumers’ willingness to use such technologies.

It is important to note that the distinction between chat-based AI and medical AI in this study does not rest on whether they employ a conversational interface. Rather, it reflects fundamental differences in their functional attributes and risk profiles across application scenarios. The chat-based AI examined here refers primarily to conversational AI products whose core functions center on everyday conversation, entertainment, and emotional companionship (Liang and Zhang, 2026; Zhang et al., 2026). Their value proposition is oriented toward relational and emotional support, and their use contexts are characterized by low risk and low professional thresholds. Medical AI, in contrast, spans a range of product forms, including online consultations, diagnostic tools, and surgical robots, and centers on disease diagnosis, treatment decision-making, and health management. These systems typically operate under conditions of high risk, high professional barriers, and high consequence sensitivity (Lai et al., 2026). It is true that some forms of medical AI, such as online consultations, resemble chat-based AI in their interactive format, as both involve human–machine dialogue (Lima et al., 2024). Nevertheless, they differ sharply in information sensitivity and potential consequences. Online medical consultations involve symptom descriptions and medical advice, and their risk profile is far higher than that of everyday emotional chat. Moreover, online consultations constitute only one subcategory of medical AI and do not capture the broader contextual characteristics of the field. Accordingly, this study treats chat-based AI and medical AI as two application types with substantial contextual heterogeneity.

IS success models generally define IS success as a construct for assessing the continued use of a system, although its specific meaning varies with the type of system (Sokro et al., 2025). In chat-based and healthcare contexts, operationalizing IS success for AI products is particularly critical. This study adopts “willingness to use” as the measure of IS success for AI products for the following reasons. First, willingness to use can be viewed as a foundational and reasonably comprehensive indicator of consumer acceptance of a specific system. According to the quality dimensions theory proposed by Salam and Farooq (2026), system quality constitutes the foundational level of perceived quality, reflecting users’ perceptions of a product’s usability and operability. Only after this level is satisfied do users proceed to evaluate information quality and service quality. Willingness to use thus corresponds closely to this foundational level of perceived quality. Second, constructs such as “behavioral intention,” “adoption intention,” and “acceptance” typically encompass multiple dimensions—including consumer cognition, emotion, and behavior—making it difficult to identify the specific level of perceived quality they capture or the level of user needs they reflect (Liu, 2025; Zhang and Yu, 2025). “Usage behavior,” by contrast, often involves higher-level elements such as social and personalization aspects rather than being confined to basic use; as a dependent variable, it would therefore be too broad and lack focus. Accordingly, this study adopts “willingness to use” as the more appropriate dependent variable. The following hypotheses are proposed:

*H1a:* Consumers’ AI literacy is positively correlated with their willingness to use chat-based AI. The higher the AI literacy, the greater consumers’ willingness to use chat-based AI.

*H1b:* Consumers’ AI literacy is positively correlated with their willingness to use medical AI. The higher the AI literacy, the greater the consumers’ willingness to use medical AI.

### 2.3 A competitive perspective: a parallel mediating model of perception of the AI use for social good and perceived AI risk

Artificial intelligence literacy, as the core competency for individuals to adapt to the technological environment in the intelligent era, fundamentally shapes consumers’ cognitive framework and behavioral patterns regarding AI technology. However, inconsistent findings in past research regarding the impact of AI literacy suggest that the relationship between AI literacy and technology adoption is highly complex and context-dependent, requiring analysis within a systematic theoretical framework. The Value-based Adoption Model provides an integrative perspective for examining this issue. Proposed by Kim et al. (2007), the model’s core logic is that users act as “value maximizers.” Compared to the early Technology Acceptance Model (TAM), which emphasized instrumental rationality dimensions such as “perceived usefulness” and “perceived ease of use,” the Value-based Adoption Model highlights the mediating role of “perceived value” (Vidyanata, 2022), which enables a more comprehensive account of the emotional, cognitive, and risk assessment processes involved in users’ technology acceptance. Prior research conceptualizes perceived value as a holistic evaluation formed by weighing benefits against costs (Kim et al., 2008). Consumers’ perceptions of AI value are inherently multifaceted and heterogeneous: they may appreciate AI for enhancing convenience and social welfare, while simultaneously remaining alert to privacy intrusions and ethical concerns (Lin et al., 2026). Given this complexity, broad measures of perceived value are ill-suited to capturing the inherent contradictions in consumer psychology. To address this, we decompose the overarching perceived value into two relatively distinct dimensions—gains and costs. Importantly, this decomposition does not abandon the trade-off logic central to VAM; rather, it translates that logic from an implicit overall evaluation into an explicit dual-path framework, where both components jointly inform consumers’ overall value judgment. In doing so, we not only preserve the theoretical core of VAM regarding cost–benefit comparisons but also extend it into an analytical model that accommodates competing psychological pathways, thereby offering a clearer explanation of how AI literacy shapes consumers’ willingness to use AI.

From the “gain” perspective based on the Value-based Adoption Model, user benefits during technology adoption are regarded as a key dimension influencing behavioral intentions. The value of artificial intelligence plays out at two levels. At the micro level, AI assists the public in everyday settings—healthcare, transportation, and entertainment—thereby improving individuals’ quality of life. At the macro level, it generates broader social benefits, particularly in addressing public issues such as poverty alleviation, climate governance, and disaster prevention (Foffano et al., 2023). Research suggests, for instance, that AI could contribute to achieving roughly 79% of the Sustainable Development Goals (Vinuesa et al., 2020). In light of this, we adopt “perceived social good” as our operational measure of “perceived gains.” This concept refers to individuals’ subjective appraisal of AI’s social value, capturing their dual consideration of its technical utility and its societal impact (Shi et al., 2020). Importantly, this construct captures the public’s core evaluation of AI’s positive outcomes through two complementary pathways—gains at the individual level and gains for society as a whole. This dual structure aligns closely with the “gains” dimension in the VAM, both conceptually and operationally. Correspondingly, our measurement items are designed to directly tap into individuals’ perceived social benefits, thereby ensuring coherence between the definition and its empirical indicators.

Previous research has preliminarily revealed a positive association between AI literacy and perceived social good. For example, Labrague et al. (2023) pointed out that AI literacy is a key antecedent variable shaping individuals’ perceptions of technology’s societal value. Individuals with higher literacy are better equipped to systematically and accurately identify the multi-level benefits brought by artificial intelligence. Furthermore, an empirical study by Alessandro et al. (2024) indicated that consumers with insufficient AI literacy often focuses solely on the immediate convenience of technology in personal life, while struggling to fully recognize AI’s potential contributions in public affairs and social governance dimensions such as environmental monitoring and disaster early warning (Alessandro et al., 2024). Thus, this study posits that higher consumers’ AI literacy leads to a stronger understanding of AI and, consequently, more positive perceptions of the social welfare generated by AI applications. Furthermore, existing research confirms the positive predictive role of individuals’ perceived social welfare on their behavioral intentions. For example, Alessandro et al. (2024) found that consumers’ perception of the social benefits of medical AI not only partially mitigates their concerns about technological risks but also significantly enhances their willingness to adopt such technologies. A study on chatbots indicated that users’ perception of welfare-oriented functions—such as “lowering communication barriers” and “alleviating loneliness”—directly translates into sustained, high-frequency usage behavior (Baek et al., 2024). Based on this, the following hypothesis is proposed:

*H2a:* Perceived social good mediates the relationship between AI literacy and the consumers’ willingness to use chat-based AI. Higher AI literacy is associated with higher perceived social good; higher perceived social good is associated with higher willingness to use chat-based AI.

*H2b:* Perceived social good mediates the relationship between AI literacy and the consumers’ willingness to use medical AI. Higher AI literacy is associated with higher perceived social good; higher perceived social good is associated with higher willingness to use medical AI.

From the “cost” perspective based on the Value-based Adoption Model, users face costs and sacrifices during the technology adoption process. According to behavioral economics perspectives, such costs can be categorized into two main types: monetary and non-monetary (Thaler, 1985). Monetary costs primarily involve users’ perception and evaluation of the actual payment price, while non-monetary costs encompass time, effort, and other intangible expenditures incurred during the use of the technology (Kim et al., 2007). For example, research indicated that non-monetary costs, such as psychological costs, significantly influence users’ perception of overall costs, thereby affecting their adoption willingness and behavioral decisions (Cronin et al., 1997). In the current development and practical context of artificial intelligence technology, the monetary costs perceived by consumers are minimal. Consumer concerns are more concentrated on non-monetary risk dimensions such as data security, algorithmic ethics, and social equity. Therefore, this study operationalizes “perceived AI risk” as a measure of perceived costs or negative factors, aiming to capture consumers’ subjective assessment of AI’s potential negative impacts across cognitive, emotional, and value dimensions. Furthermore, perceived AI risk—as consumers’ assessment of the adverse consequences of AI use—essentially constitutes a cognitive representation of “potential loss.” Previous research has shown that perceived risk is a key negative factor that undermines public trust in and willingness to use AI (Li et al., 2025). This aligns closely with the assessment logic in cognitive psychology, which views risk as a potential sacrifice or loss; therefore, treating it as an operationalized variable within the “cost” dimension is appropriate both theoretically and empirically.

Research indicated that AI literacy can foster comprehensive understanding and cognition of artificial intelligence, thereby reducing consumer fear and risk concerns regarding AI (Cengiz and Peker, 2025). However, some studies suggested the opposite perspective: Yao et al. (2025) found in their research on medical AI that users with higher AI literacy actually demonstrated lower levels of trust in medical AI. A study on college students’ ChatGPT usage behavior indicated that individuals who understand natural language processing mechanisms are more likely to detect the “hallucination” phenomenon in generative AI, thereby recognizing the accuracy risks of its output content (Al-Abdullatif and Alsubaie, 2024). Therefore, this study posits that individuals with higher AI literacy are more likely to discern the “black box” nature of AI systems, algorithmic biases, and their potential risks. This awareness can lead to negative cognitive and emotional responses, such as skepticism and anxiety, ultimately resulting in a higher overall risk perception. Furthermore, extensive empirical research has revealed a consistent negative correlation between individuals’ risk perceptions of technology and their behavioral intentions. For instance, Orsot-Dessi et al. (2023) found in their study on autonomous vehicles that perceived risk significantly inhibited consumer adoption intentions. Tseng (2025) further noted that perceived risk, as a key antecedent in the adoption process of digital technologies, indirectly weakens adoption willingness by inducing consumer anxiety—that is, higher risk perception levels lead to greater individual anxiety, which in turn significantly reduces adoption intentions. Based on this, the following hypothesis is proposed:

*H3a:* Perceived AI risk mediates the relationship between AI literacy and the consumers’ willingness to use chat-based AI. Higher AI literacy is associated with higher perceived AI risk; higher perceived AI risk is associated with lower willingness to use chat-based AI.

*H3b:* Perceived AI risk mediates the relationship between AI literacy and the consumers’ willingness to use medical AI. Higher AI literacy is associated with higher perceived AI risk; higher perceived AI risk is associated with lower willingness to use medical AI.

Based on the above, this study constructs a competitive parallel mediation model with AI literacy as the independent variable, perceived social good and perceived AI risk as parallel mediating variables, and willingness to use AI products as the outcome variable. Within this model, the two mediation paths exhibit opposite directions of influence. On one hand, higher AI literacy correlates with stronger perceived social good and greater willingness to use AI. On the other hand, higher AI literacy correlates with heightened perceived AI risk and reduced willingness to use AI. This model not only systematically elucidates the dual-pathway mechanism through which AI literacy influences consumers’ willingness to use AI but also provides a theoretical pathway to reconcile divergent findings regarding the directionality of AI literacy’s effects in existing research (Figure 1).

**Figure 1:**
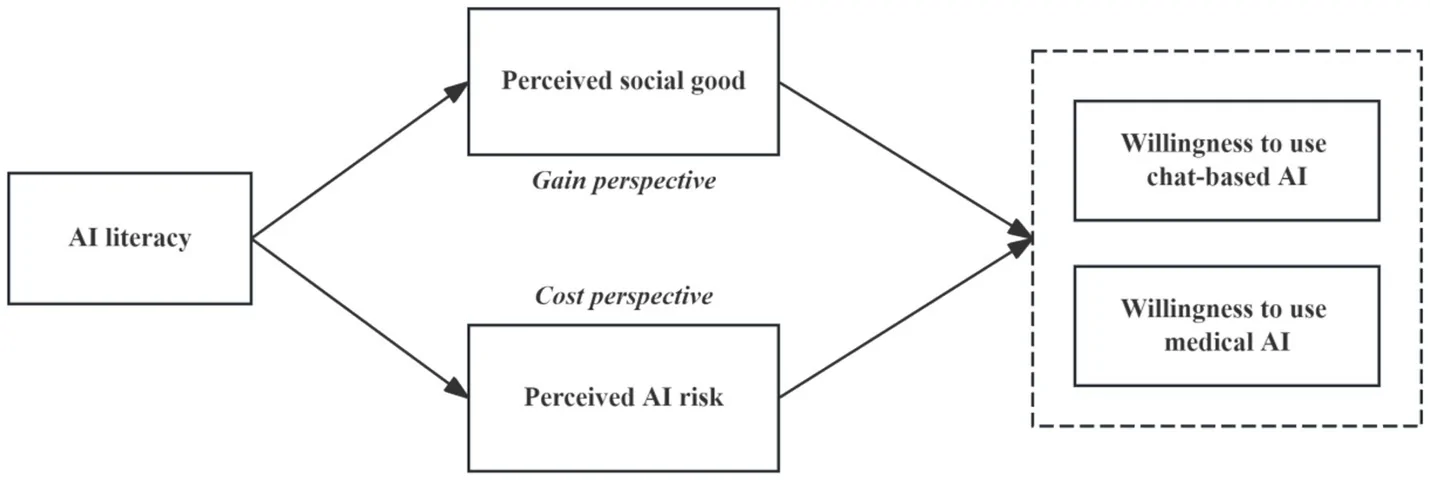
Parallel mediating model of AI literacy on willingness to use AI.

## 3 Materials and methods

### 3.1 Participants

This study distributed questionnaires via the Credamo platform and utilized the platform’s randomization program to ensure that questionnaires were randomly assigned to participants. All participants are from China. After excluding questionnaires with abnormal response times and invalid responses, a total of 441 valid questionnaires were obtained. Among the sample, 27% (*N* = 119) were male, and 73% (*N* = 322) were female. The average age of the participants was 37.86 (Table 1).

**Table 1:** Sample characteristics (*N* = 441).

Demographic variables		Frequencies (percentages)
Age	10–19	3 (0.7%)
	20–29	116 (26.3%)
	30–39	264 (59.9%)
	40–49	45 (10.2%)
	50–59	12 (2.7%)
	≥60	1 (0.2%)
Gender	Male	119 (27%)
	Female	322 (73%)

### 3.2 Measure

Informed consent was obtained from the participants in this study, and they were informed that their responses would be kept strictly confidential. All methods were performed in accordance with the Declaration of Helsinki. This study was approved by the Ethics Committee at the authors’ institution (approval number: JNUSJC2026113). All scales used in this study were translated from their original English versions and administered in Chinese. The translation and back-translation procedure was strictly followed (Brislin, 1970). First, two researchers proficient in both Chinese and English independently translated the original scales into Chinese; after comparing their translations and resolving discrepancies through discussion, they finalized an initial Chinese version. Next, a third researcher, who had no prior exposure to the original scales, back-translated the Chinese draft into English. A comparison revealed that the back-translated version was highly consistent with the original scales at both the semantic and conceptual levels. Additionally, we used machine translation software to back-translate the Chinese version of the scales into English; the resulting items were largely consistent with the original items, further supporting translation equivalence. In subsequent measurements, all scales demonstrated good reliability within the study sample (Cronbach’s α = 0.79–0.97), indicating that the Chinese versions were suitable for the Chinese participants in the present study.

#### 3.2.1 AI literacy

We measured the AI literacy using an 18-item scale (Schiavo et al., 2024). The participants were asked to indicate their level of agreement with a list of statements (1 = “Strongly disagree,” 7 = “Strongly agree”). A sample item is as follows: “I can evaluate the pros and cons of using artificial intelligence” (α = 0.89).

#### 3.2.2 Perceived social good

“Perceived social good” refers to the public’s comprehensive, subjective assessment of the benefits generated by the application of artificial intelligence at the societal level and the extent of those benefits. We measured the perceived social good using a 5-item scale (Chai et al., 2021). The participants were asked to indicate their level of agreement with a list of statements (1 = “Strongly disagree,” 7 = “Strongly agree”). A sample item is as follows: “Artificial intelligence can benefit society” (α = 0.79).

#### 3.2.3 Perceived AI risk

We measured the perceived AI risk using a 14-item scale (Gupta et al., 2023). The participants were asked to indicate their level of agreement with a list of statements (1 = “Strongly disagree,” 7 = “Strongly agree”). A sample item is as follows: “I believe that the use of artificial intelligence carries the risk of putting more people out of work.” (α = 0.97).

#### 3.2.4 Willingness to use AI

We measured consumers’ willingness to use AI using an 8-item scale (Yue and Li, 2023). The participants were asked to indicate their level of agreement with a list of statements (1 = “Strongly disagree,” 7 = “Strongly agree”). A sample item is as follows: “I would recommend medical AI/ chat-based AI to others.” (medical AI α = 0.86; chat-based AI α = 0.81).

## 4 Results

### 4.1 Preliminary analysis

Before testing our hypothesis, we conducted a preliminary analysis. Harman’s single-factor test was used to determine the variance for the single-factor solution (variance = 24.62%), which indicated that there is no serious common method variance (CMV) in this study (Podsakoff and Organ, 1986). Multicollinearity diagnostics indicated that the variance inflation factors (VIFs) for all predictors ranged from 1.009 to 1.815, well below the conventional threshold of 10, suggesting that multicollinearity was not a concern in this study. The descriptive statistics and correlation matrix pertaining to our focal variables are shown in Table 2. In our subsequent analysis, we used Model 4 of the PROCESS plugin for SPSS and the bootstrap method with structural equation modeling (SEM) in AMOS to test mediation effects.

**Table 2:** Descriptive statistics and intercorrelations among variables (*N* = 441).

Variables	*M*	*SD*	1	2	3	4	5
AI literacy	5.83	0.54	1				
Perceived social good	5.90	0.70	0.66^**^	1			
Perceived AI risk	4.48	1.43	0.10^*^	0.08	1		
Willingness to use medical AI	5.42	1.01	0.13^**^	0.10^*^	−0.42^**^	1	
Willingness to use chat-based AI	5.83	0.78	0.35^**^	0.41^**^	−0.18^**^	0.37^**^	1

*N* = 441. M = mean; SD = standard deviation. ^*^p < 0.05, ^**^p < 0.01.

Subsequently, we conducted a regression analysis to test H1a and H1b. AI literacy was positively correlated with consumers’ willingness to use chat-based AI (b = 0.50, *p* < 0.001). The effect persisted after controlling for gender and age (b = 0.50, *p* < 0.001). AI literacy was positively correlated with consumers’ willingness to use medical AI (b = 0.23, *p* = 0.009). The effect persisted after controlling for gender and age (b = 0.23, *p* = 0.01). Thus, H1a and H1b were supported.

### 4.2 Model testing

H2a posited that perceived social good mediates the relationship between AI literacy and consumers’ willingness to use chat-based AI. We used model 4 of the PROCESS macro in SPSS for this analysis. As indicated in Table 3, after controlling for participants’ gender and age, the predictive effect of AI literacy on perceived social good was significant (b = 0.86, *p* < 0.001), and the predictive effect of perceived social good on consumers’ willingness to use chat-based AI was significant (b = 0.36, *p* < 0.001). As indicated in Table 4, the mediation analysis results revealed that the direct effect was significant (b = 0.22, 95% confidence interval (CI) = [0.06, 0.38]), and the indirect effect was significant (b = 0.32, 95% confidence interval (CI) = [0.19, 0.45]). The mediating effect was a partial mediating effect. Therefore, H2a was supported.

**Table 3:** Regression analysis of the mediation model (*N* = 441).

Model/variable	b	SE	t	*p*
Mediator variable (perceived social good) model				
Constant	1.04	0.60	1.74	0.08
Gender	0.09	0.06	1.67	0.10
Age	0.00	0.00	−0.38	0.71
AI literacy	0.86^***^	0.05	18.54	< 0.001
Mediator variable (perceived AI risk) model				
Constant	3.66^*^	1.63	2.25	0.05
Gender	−0.01	0.15	−0.08	0.94
Age	0.00	0.00	−0.49	0.62
AI literacy	0.26^*^	0.13	2.08	0.04
Dependent variable (willingness to use chat-based AI) model				
Constant	2.66^**^	0.70	3.35	0.001
Gender	0.04	0.07	0.59	0.56
Age	0.00	0.00	0.40	0.69
AI literacy	0.22^**^	0.08	2.66	0.008
Perceived social good	0.36^***^	0.06	5.81	< 0.001
Perceived AI risk	−0.12^***^	0.02	−5.17	< 0.001
Dependent variable (willingness to use medical AI) model				
Constant	5.17^***^	1.04	4.98	< 0.001
Gender	0.09	0.10	0.96	0.34
Age	0.00	0.00	−0.29	0.77
AI literacy	0.27^*^	0.11	2.54	0.012
Perceived social good	0.05	0.08	0.55	0.58
Perceived AI risk	−0.30^***^	0.03	−10.05	< 0.001

Unstandardized regression coefficients are reported. Bootstrapping sample size = 5,000. ^*^p ≤ 0.05, ^**^p ≤ 0.01, ^***^p ≤ 0.001.

**Table 4:** Bootstrapping analysis of the mediation model (*N* = 441).

Model/effect		Effect	SE	BootLLCI	BootULCI
Dependent variable (willingness to use chat-based AI) model					
Direct effect		0.22	0.08	0.06	0.38
Indirect effect	Total	0.28	0.07	0.16	0.42
	Perceived social good	0.32	0.07	0.19	0.45
	Perceived AI risk	−0.03	0.02	−0.07	−0.003
Total effect		0.50	0.07	0.37	0.63
Dependent variable (willingness to use medical AI) model					
Direct effect		0.27	0.11	0.06	0.48
Indirect effect	Total	−0.04	0.08	−0.21	0.11
	Perceived social good	0.04	0.07	−0.11	0.17
	Perceived AI risk	−0.08	0.04	−0.16	−0.01
Total effect		0.23	0.09	0.06	0.41

H2b posited that perceived social good mediates the relationship between AI literacy and consumers’ willingness to use medical AI. We used model 4 of the PROCESS macro in SPSS for this analysis. As indicated in Table 3, after controlling for participants’ gender and age, the predictive effect of AI literacy on perceived social good was significant (b = 0.86, *p* < 0.001), and the predictive effect of perceived social good on consumers’ willingness to use medical AI was not significant (b = 0.05, *p* = 0.58). As indicated in Table 4, the mediation analysis results revealed that the direct effect was significant (b = 0.27, 95% confidence interval (CI) = [0.06, 0.48]), and the indirect effect was not significant. Therefore, H2b was not supported.

H3a posited that perceived AI risk mediates the relationship between AI literacy and consumers’ willingness to use chat-based AI. We used model 4 of the PROCESS macro in SPSS for this analysis. As indicated in Table 3, after controlling for participants’ gender and age, the predictive effect of AI literacy on perceived AI risk was significant (b = 0.26, *p* = 0.04), and the predictive effect of perceived AI risk on consumers’ willingness to use chat-based AI was significant (b = −0.12, *p* < 0.001). As indicated in Table 4, the mediation analysis results revealed that the direct effect was significant (b = 0.22, 95% confidence interval (CI) = [0.06, 0.38]), and the indirect effect was significant (b = −0.03, 95% confidence interval (CI) = [−0.07, −0.003]). The mediating effect was a partial mediating effect. Therefore, H3a was supported. Moreover, within this parallel mediation model, the positive mediating effect of perceived social good is stronger than the negative mediating effect of perceived AI risk.

H3b posited that perceived AI risk mediates the relationship between AI literacy and consumers’ willingness to use medical AI. We used model 4 of the PROCESS macro in SPSS for this analysis. As indicated in Table 3, after controlling for participants’ gender and age, the predictive effect of AI literacy on perceived AI risk was significant (b = 0.26, *p* = 0.04), and the predictive effect of perceived AI risk on consumers’ willingness to use medical AI was significant (b = −0.30, *p* < 0.001). As indicated in Table 4, the mediation analysis results revealed that the direct effect was significant (b = 0.27, 95% confidence interval (CI) = [0.06, 0.48]), and the indirect effect was significant (b = −0.08, 95% confidence interval (CI) = [−0.16, −0.01]). The mediating effect was a partial mediating effect. Therefore, H3b was supported.

In addition, we tested the parallel mediation model using AMOS 24.0 to verify the robustness of the results. We included AI literacy as the independent variable, perceived AI risk and perceived social good as mediating variables, willingness to use chat-based AI and willingness to use medical AI as the two dependent variables, and age and gender as control variables in the model. The model demonstrated good fit: *χ*^2^ = 3.136, *df* = 4, *p* = 0.535, *χ*^2^/*df* = 0.784, CFI = 1.000, RMSEA = 0.000, 90% CI [0.000, 0.065], pclose = 0.873, making it appropriate for structural equation modeling.

Results showed that AI literacy exerted significant positive direct effects on both willingness to use chat-based AI (*β* = 0.15, *p* = 0.008) and willingness to use medical AI (*β* = 0.15, *p* = 0.011). In chat scenarios, both perceived AI risk (*β* = −0.22, *p* < 0.001) and perceived social good (*β* = 0.33, *p* < 0.001) functioned as significant parallel mediators. In medical scenarios, only perceived AI risk served as a significant mediator (*β* = −0.43, *p* < 0.001), whereas the mediating path through perceived social good was not statistically significant (*β* = 0.03, *p* = 0.585). The control variables age and gender showed no significant associations with either outcome.

## 5 Discussion

The pervasive integration of artificial intelligence across societal sectors has been paralleled by a dual narrative of remarkable technological efficacy and profound societal controversy. Against this backdrop, exploring how AI literacy empowers consumers’ acceptance and adoption of intelligent technologies has become a critical topic in current human-machine communication research. Existing literature presents conflicting conclusions regarding the impact of AI literacy on consumers’ cognitive attitudes and behavioral intentions, with most studies confined to single application contexts and lacking systematic examination of cross-domain heterogeneity. Therefore, this study adopts the Value-based Adoption Model, selecting chat scenarios and medical scenarios as contrasting contextual domains. It systematically evaluates the pathways through which AI literacy influences consumers’ willingness to use AI products and further reveals the underlying mechanisms. This study aims to provide theoretical explanations for the conflicting conclusions in existing literature on AI literacy and technology acceptance, thereby deepening the understanding of how AI literacy empowers consumers’ adoption of intelligent technologies.

### 5.1 Research findings

First, the primary findings of this study indicate that consumers’ AI literacy is positively associated with their willingness to use AI. Specifically, the higher an individual’s level of AI literacy, the stronger their willingness to use AI. This finding aligns with the conclusions of most previous empirical studies, confirming the positive association between AI literacy and consumers’ cognition, attitudes, and behavioral tendencies toward artificial intelligence technology (Si, 2025; Zhang D. et al., 2025). A plausible explanation of this positive association is that higher AI literacy is associated with consumers’ moving beyond blind reverence or irrational fear toward technology. Instead, higher AI literacy is associated with a more dialectical perspective on the functional boundaries and societal implications of AI products (Archambault et al., 2025). Users with higher AI literacy can more clearly understand the operational logic of AI and its applicable scenarios in daily life (such as emotional companionship, knowledge acquisition, and medical aids), thereby more readily forming positive usage expectations and behavioral tendencies (Knoth et al., 2024).

Second, this study provides preliminary evidence that AI literacy has a double-edged impact on consumers’ willingness to use AI products. In chat scenarios, AI literacy indirectly influences consumers’ willingness to use such products through their perceived social good. This finding aligns with existing research, which indicates that AI literacy typically has a positive impact on users’ cognition and behavior in human-computer interaction contexts (Dai, 2024). For example, research has found that AI literacy helps enhance learners’ self-efficacy in AI learning and reduce their anxiety, ultimately increasing their willingness to engage with AI (Zhang Q. et al., 2025). However, AI literacy can also negatively affect consumers’ willingness to use AI products through their perceived risk. This negative effect is relatively rare in the existing literature but is receiving increasing attention. For instance, a study by Li et al. (2026) showed that consumers with higher AI literacy actually demonstrated lower acceptance of AI technology. Thus, AI literacy indeed exerts a double-edged impact on consumers’ willingness to adopt AI products. This dual effect has received empirical support in a previous study focusing on AI news. Increased AI literacy may either strengthen readers’ preference for algorithms or intensify their aversion to them, leading to polarized evaluations of the credibility of AI news (Kim and Ryoo, 2026). This study corroborates the existence of this pattern from the perspective of AI product consumption.

Furthermore, in medical AI scenarios, perceived AI risk plays a negative mediating role, while perceived social good does not mediate the relationship. This phenomenon can also be interpreted as a suppression pattern (Tzelgov and Henik, 1991). Specifically, the higher consumers’ AI literacy, the greater their perception of AI risk, and this perception of risk will negatively affect consumers’ willingness to use medical AI. The RAIDUA model, which is highly insightful for research on human-computer interaction in the fields of marketing and tourism, highlights how consumers’ privacy concerns influence their usage behavior. Related work further shows that stronger privacy concerns significantly reduce consumers’ willingness to accept and use AI-enabled devices (Salam et al., 2026b). Perceived risks such as privacy concerns thus play a non-negligible role in consumer decision-making. Consumers with higher AI literacy, in particular, tend to be more aware of the privacy and ethical risks associated with AI products (Barthwal et al., 2025). Efforts to foster AI literacy should therefore include managing and guiding these perceived risks. Only through appropriate risk management and behavioral guidance can consumers be encouraged to use AI responsibly. The parallel mediation model confirmed in this research effectively integrates conflicting conclusions from prior studies regarding AI literacy’s impact: Some studies emphasize AI literacy’s positive role in fostering favorable perceptions and usage willingness, while others suggest potential negative effects (Weikang et al., 2025). By revealing the coexistence and interplay of two parallel mediating pathways, this study provides an integrative explanatory framework for these seemingly contradictory conclusions.

Third, this study finds that the influence of AI literacy on consumers’ willingness to use AI exhibits different patterns across various contexts. In chat-based AI scenarios, the perceived social good and perceived AI risk play competing dual mediating roles. Specifically, the positive mediating effect of the perceived social good is stronger than the negative mediating effect of perceived AI risk. However, in medical AI scenarios, only perceived AI risk plays a mediating role. This difference may stem from the profound impact of scenario attributes on individual technology acceptance. Specifically, chat scenarios are characterized by both low risk and high demand for personalization; users’ adoption of this technology is often driven more by a pursuit of convenience (Klaus and Zaichkowsky, 2022). Individuals with higher AI literacy can leverage their solid technical skills and functional understanding to more efficiently translate AI capabilities into immediate experiential value, thereby enhancing their willingness to use the technology. In contrast, in medical scenarios, because medical decisions directly relate to life and health, individuals exhibit greater sensitivity to perceived risks (Said et al., 2025). Consumer concerns regarding technological uncertainty, the risk of misdiagnosis, and privacy issues may overshadow the rational judgment derived from high AI literacy, thereby limiting the influence of perceived well-being on adoption willingness.

### 5.2 Theoretical implications

First, this study extends the applicability of the Value-based Adoption Model to the domain of AI acceptance, offering a novel theoretical lens for understanding consumer adoption behavior. Traditional models primarily focus on users’ perceptions of the cost-effectiveness of new technologies, whereas our framework incorporates AI literacy and usage contexts as key factors. To capture the dual-edged impact of AI literacy, we decompose perceived value into two opposing dimensions: perceived social good and perceived AI risk. This operational definition addresses the “influence paradox” of AI literacy observed in previous studies (Kim and Ryoo, 2026). By adopting a parallel mediation model, this study elucidates the process by which AI literacy shapes consumers’ willingness to use AI through a dual-path mechanism, thereby shifting the research agenda from “whether AI literacy is important” to “how it functions through competing psychological pathways.”

Second, we developed and conducted a preliminary test of a theoretical model that specifies the mediating mechanisms through which AI literacy translates into usage intention, using an adult consumer sample rather than the student or educational samples common in prior work. In this model, AI literacy serves as the independent variable, willingness to use AI as the dependent variable, and perceived social good and perceived AI risk as parallel mediators. This design responds to an unresolved theoretical question in the AI literacy literature: whether the mixed findings reflect unmeasured opposing mechanisms rather than unstable direct effects. Our findings reveal that, on one hand, AI literacy positively affects consumers’ willingness to use chat-based AI by perceived social good. On the other hand, it negatively affects this willingness by perceived AI risks. Notably, the positive mediating effect of perceived social good outweighs the negative mediating effect of perceived AI risk. This discovery demonstrates that the net effect of AI literacy is not a single homogeneous influence but the result of a dual-pathway offsetting mechanism, theoretically reconciling the existing debate between the “promotion theory” and “inhibition theory” of AI literacy and offering a mechanistic explanation for previously inconsistent results.

Finally, this study proposes a perspective on scenario comparison to reveal how the influence of AI literacy on consumers’ usage willingness varies across different scenarios. In chat-based AI scenarios, AI literacy significantly impacts willingness to use through dual-path mediation. In medical AI scenarios, AI literacy exerts a significant negative effect on consumers’ willingness to use medical AI through their perception of AI risks. At the same time, AI literacy does not have a positive effect on consumers’ willingness to use medical AI through the perceived social good. These findings advance our understanding of AI adoption, underscoring the extent to which adoption processes are embedded in specific use scenarios. Future research should, first, adopt a scenario-based perspective by systematically comparing application scenarios—such as healthcare, conversational agents, autonomous driving, smart home technologies, and psychological counseling—to clarify the distinct mechanisms shaping the public’s use of various AI products. Second, researchers should focus on the differences between various types of AI products, combining theoretical analysis with empirical investigation to uncover the mechanisms that generate scenario-specific differences. Such efforts would deepen theoretical accounts of the public’s scenario-sensitive use of AI.

### 5.3 Practical implications

Our research holds significant practical implications for advancing AI literacy to promote informed adoption of intelligent technologies among adult consumers. Firstly, it underscores the importance of systematically cultivating consumers’ AI literacy. Findings confirm that AI literacy exerts a significant positive influence on consumers’ perceived social good, indirectly boosting willingness to adopt such technologies through this pathway. This indicates that enhancing consumers’ AI literacy serves as an effective lever for increasing acceptance of intelligent technologies. Therefore, governments, educational institutions, and various social organizations should collaborate to establish an AI literacy education system. For instance, for youth groups, AI literacy education can be integrated into basic education curricula to cultivate their ability to critically understand intelligent technologies. For middle-aged and elderly individuals, as well as those with lower educational attainment, community science outreach and public lectures can lower the technical cognitive threshold, helping them develop a positive and rational cognitive framework regarding AI technology.

Secondly, implement scenario-differentiated AI literacy cultivation strategies. The scenario comparison analysis in this study indicates that the mechanisms of AI literacy vary significantly across different types of AI products. These findings necessitate the development of scenario-specific and precision-targeted strategies for AI literacy. For instance, regarding chat-based AI, consumer outreach should emphasize demonstrating its enhancement of daily convenience, reinforcing positive consumer perceptions through case-based teaching and experiential learning. For medical AI, AI literacy cultivation should prioritize ethical risk education and rational cognition building. This guides consumers toward forming prudent, balanced judgments between risk awareness and value perception, preventing irrational over concern or blind trust stemming from information asymmetry. In addition, students should be encouraged to engage in collaborative reflection through platforms provided by schools, social organizations, and other institutions (Salam et al., 2026a). Such dialogue and exchange of experience can enhance their critical thinking and foster a deeper, more comprehensive understanding of various artificial intelligence products.

Finally, the dual-pathway mechanism identified in this study underscores the need to emphasize risk management in the cultivation of AI literacy. Research confirms that AI literacy can indirectly diminish consumers’ willingness to use AI by amplifying risk perception. This finding cautions us that fostering consumer AI literacy must proceed in tandem with transparent risk communication mechanisms. On one hand, science communication efforts should maintain an objective and balanced stance. They must accurately convey the potential risks of AI technology while systematically explaining progress in building industry regulatory frameworks and technical ethical standards, thereby helping consumers develop a rational understanding. On the other hand, relevant enterprises and regulatory authorities should improve information disclosure and risk warning systems for AI products. By enhancing technological transparency, they can reduce consumer fear of the unknown. These combined measures will effectively mitigate the negative impact of risk perception on consumers’ willingness to use AI, while concurrently enhancing consumers’ literacy.

### 5.4 Limitations and future directions

This study has the following limitations. First, the scope of scenarios covered is limited. Future research could expand to more diverse AI application scenarios to validate the universality of the findings. This study focuses on a comparative analysis between two typical applications—medical AI and chat-based AI—which reveals the impact of scenario heterogeneity to some extent. However, it has not yet incorporated emerging scenarios with higher ethical sensitivity and risk controversies, such as autonomous driving and AI resurrection. Future research should broaden the range of scenarios examined to investigate the mechanisms through which AI literacy influences consumers’ willingness to adopt AI across different technological attributes, usage contexts, and levels of societal acceptance. Such an expansion would deepen our understanding of the boundaries of AI literacy’s effectiveness.

Second, with respect to control variables, this study followed common practice in related research by controlling for gender and age but not for other demographic characteristics such as educational attainment or occupational background. Although this choice simplifies the model and keeps the focus on key mechanisms, it may limit the external validity of the findings. Future research could incorporate a broader set of demographic controls. With respect to sample composition, owing to how data were collected through the Credamo survey platform, the sample is somewhat gender-imbalanced, with female participants substantially outnumbering male participants. The data for this study were drawn from a single online sample pool, whose users may exhibit systematic differences from the general population in terms of age, educational attainment, and usage habits. Therefore, the study’s conclusions may be subject to selection bias, and caution should be exercised when generalizing these findings. Future studies might improve the sample balance by combining offline questionnaire distribution with recruitment across multiple platforms. In addition, consumer behavior related to the use of AI in healthcare may be moderated or confounded by health-related factors such as individuals’ current health status and medical history. Future research could therefore incorporate these individual-level characteristics into the analysis to strengthen the explanatory power of the theoretical model.

Third, this study employed a questionnaire survey method, with all data derived from respondents’ self-reports. While this method facilitates the direct collection of individuals’ subjective perceptions and attitudes toward artificial intelligence technology, it also has certain limitations. On the one hand, respondents may not be able to truthfully and accurately reflect their internal cognition and actual behavior. On the other hand, there may still be a gap between the “willingness to use” measured via self-reports and actual technology adoption behavior. In reality, the public’s willingness does not necessarily translate into actual usage. Therefore, future research could explore the use of diverse data collection methods, such as big data mining and eye-tracking, to provide more refined empirical support for understanding the underlying mechanisms linking AI literacy to behavioral intentions.

Finally, regarding research design, this study employed a cross-sectional design, which limits valid inferences about the temporal sequence and causal relationships among variables. Future research could incorporate a longitudinal tracking or quasi-experimental design to strengthen causal identification and the robustness of conclusions. In addition, this study employed well-established scales as measurement tools; consequently, the evidence for validity relies primarily on the inherent quality of these scales, and no additional validation—such as for convergent validity or discriminant validity—was conducted, which may have a potential impact on the accuracy of variable measurement. Therefore, caution should be exercised when generalizing the study’s findings to broader contexts.

## Data Availability

The datasets presented in this study can be found in online repositories. The names of the repository/repositories and accession number(s) can be found at: https://osf.io/wvxdq/overview?view_only=129c94df3aa74086a946543ec61a981d.
